# Influence of Leaf Litter Moisture on the Efficiency of the Winkler Method for Extracting Ants

**DOI:** 10.1673/031.012.5701

**Published:** 2012-04-25

**Authors:** Thibaut D. Delsinne, Tania M. Arias-Penna

**Affiliations:** Royal Belgian Institute of Natural Sciences, Biological Evaluation Section, 29 rue Vautier, 1000 Brussels, Belgium

**Keywords:** ants of leaf litter protocol, Ecuador, Formicidae, mountain rainforest, rainfall exclusion, rapid biodiversity assessment, sampling method evaluation, Winkler extraction time

## Abstract

The Winkler extraction is one of the two fundamental sampling techniques of the standardized “Ants of the Leaf Litter” protocol, which aims to allow qualitative and quantitative comparisons of ant (Hymenoptera: Formicidae) assemblages. To achieve this objective, it is essential that the standard 48—hour extraction provides a reliable picture of the assemblages under study. Here, we tested to what extent the efficiency of the ant extraction is affected by the initial moisture content of the leaf litter sample. In an Ecuadorian mountain rainforest, the leaf litter present under rainfall—excluded and rainfall—allowed plots was collected, its moisture content measured, and its ant fauna extracted with a mini—Winkler apparatus for a 48—hour and a 96—hour period. The efficiency of the Winkler method to extract ant individuals over a 48—hour period decreased with the moisture content of the leaf litter sample. However, doubling the extraction time did not improve the estimations of the ant species richness, composition, and relative abundance. Although the moisture content of the leaf litter slightly affected the ant sampling, our results indicated that a 48—hour Winkler extraction, as recommended by the “Ants of the Leaf Litter” protocol, is sufficient to allow reliable comparisons of ant assemblages.

## Introduction

The Winkler extraction is a rapid, simple, cost-effective, and repeatable method to collect ants (Hymenoptera: Formicidae) of the leaf litter (Bestelmeyer et al. 2000; Delabie et al. 2000). This method, along with pitfall traps, constitute the fundamental sampling techniques of the standardized “Ants of the Leaf Litter” (A.L.L.) protocol (Agosti and Alonso 2000). The latter was developed to allow qualitative and quantitative comparisons of ant assemblages at different localities or over time, for use in biological evaluation and conservation, assemblage monitoring, and description of diversity patterns. In order to carry out reliable comparisons, the Winkler extraction duration should ideally be sufficient to collect all the ants present in the sample, or at least to provide a correct picture of the assemblage structure. The standard Winkler extraction of the A.L.L. protocol lasts 48 hours, but a survey of the literature shows that a large variety of extraction durations have been used, often without a justification (the extraction time ranged from 0 to 10 days or was not given; a 48—hour extraction was used in less than 50 % of the 73 studies surveyed. Supplementary details are provided in the Appendix). This diversity makes inter-study comparisons potentially challenging, especially because a very long time is often necessary to obtain a complete extraction of the ant fauna (Krell et al. 2005; Sakchoowong et al. 2007). For instance, up to 15 days were necessary to extract all ants present in leaf litter samples from temperate forests of England (Krell et al. 2005). In addition, because the Winkler method is partly based on the passive dessication of the leaf litter (Bestelmeyer et al. 2000; Krell et al. 2005), the completeness of ant extraction might be affected by the moisture content of the sample, with wetter samples requiring longer extraction times than drier ones. If it is the case, using the Winkler method to compare the ant assemblage structure among seasons, between moist and dry habitats, or even before and after a rain might be irrelevant. In this study, the leaf litter moisture of a mountain rainforest was experimentally manipulated to test the following hypotheses. First, the completeness of a 48—hour Winkler extraction, as recommended by the A.L.L. protocol, is not affected by the initial moisture content of the leaf litter sample. This result would be obtained if the ant extraction relies mainly on the disturbance of the leaf litter rather than on its passive dessication. Second, a 48—hour extraction is sufficient to obtain a reliable picture of the ant assemblage, whatever the initial moisture content of the leaf litter sample. To test this hypothesis, we compared the composition and the species relative abundance of the ant assemblages after a 48—hour and a 96—hour Winkler extraction for both dry and moist leaf litter samples.

**Figure 1.**
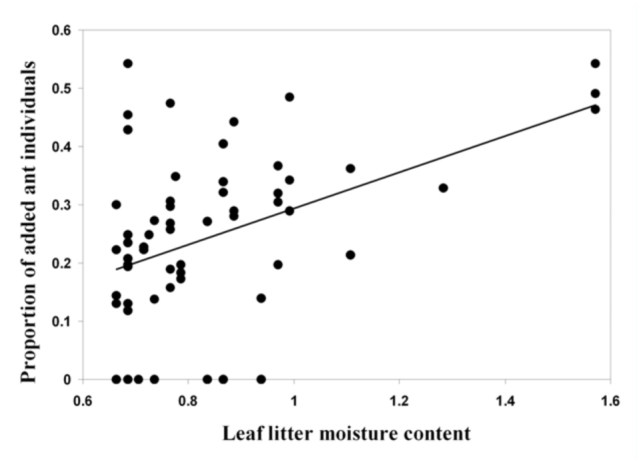
Relationship between the moisture content of the leaf litter sample and the proportion of ant individuals added by the second Winkler extraction. Only data from rainfall-excluded plots were analyzed (*n* = 60). Data from rainfall-allowed plots were not included because the moisture content of almost all these samples was maximal (Table 1 ). Both axes are arcsin square root transformed. The best—fitting equation of the regression analysis was: asinsqrt (Proportion of added individual) = -0.01 7 + 0.3 11 × asinsqrt (Leaf litter moisture); adjusted r^2^ = 0.189, *p* < 0.01. High quality figures are available online.

## Materials and Methods

The study was carried out at 2000 m a.s.l. at the “te;serva BiologíFrancisco” situated within the Eastern Cordillera of the Ecuadorian Andes, in the province of ZamoraChinchipe (3° 58' S, 79° 5' W). Vegetation corresponds to an evergreen upper montane forest (Hormeier et al. 2008). Mean annual precipitation is 2100 mm (Bendix et al. 2008). Mean temperature in the leaf litter during the experiment was 16 ^°^C (min-max: 12.7-21.5 ^°^C). Atmospheric relative humidity at 1.5 m above ground ranged from 91 to 95% during sampling.

**Figure 2.**
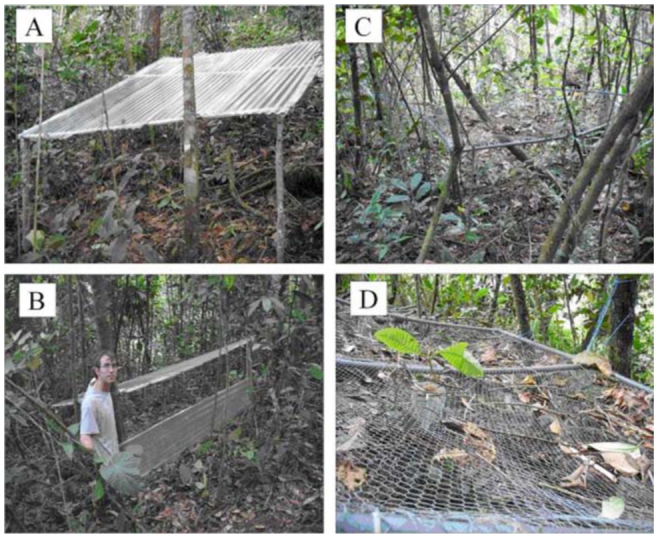
Rainfall—excluded (A, B) and rainfall-allowed (C, D) plots. High quality figures are available online.

In November 2009, six 3 ×3 m plots, spaced 2 to 20 m apart, were randomly assigned to either rainfall—excluded (*n* = 3) or rainfall—allowed (*n* = 3) plots. Rainfall exclusion was achieved by installing transparent plastic sheets at 1-1.2 m above ground. At the top side of the plot, a supplementary sheet was buried to a depth of 30 cm to keep running water from going inside. The three other sides were left open to limit any greenhouse effect. A mesh replaced the sheets at rainfall—allowed plots to exclude falling leaves but to allow rainfall inputs (Figure 2).

**Figure 3.**
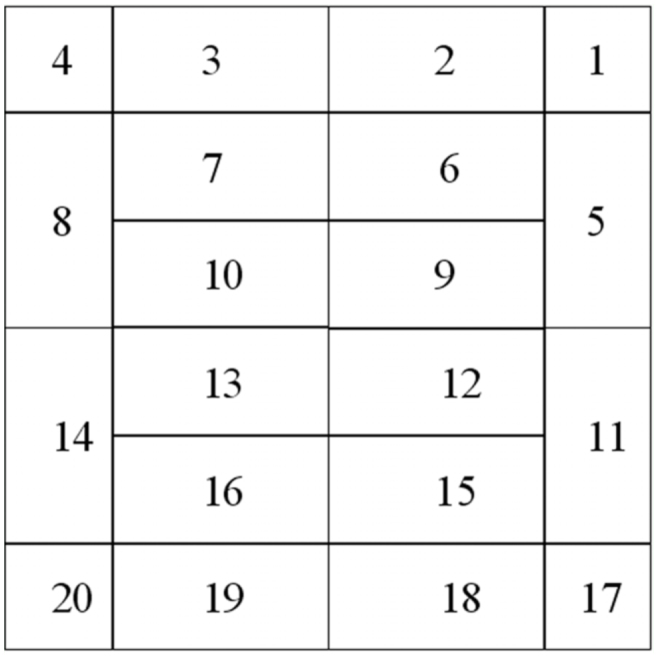
Quadrat disposition within each plot. Each plot was a 3 × 3 m square. The disposition of the quadrats was chosen in order to obtain a 50 cm border around a 4 m^2^ area. The fact that quadrats are of two different sizes (0.5 and 0.25 m^2^) is a consequence of this design. High quality figures are available online.

In May 2010, ants were collected using the Winkler method (Bestelmeyer et al. 2000). One plot of each treatment was always sampled during a single day. Sampling was carried out at least a day after significant rainfall to limit the risk of arthropods (especially small ones) sticking to the wet litter, and thus not being effectively extracted (Fisher 1996). The leaf litter present inside a 0.5 m^2^ or a 0.25 m^2^ quadrat (*n =* 16 and 4/plot, respectively) was collected and sifted (Figure 3 shows details of the quadrat disposition within each plot). The moisture content (using a Protometer Mini moisture meter, www.romus.org), volume, and weight of the sifted leaf litter were measured and its fauna was extracted with a mini—Winkler apparatus (Fisher 1996, 1998). All the extractions operated in the same room. After a 48—hour extraction, the collecting container was replaced by a new one and a second extraction was performed over a 48—hour period. No additional search for remaining ants was made after the second extraction, since such a procedure may be highly time—consuming (Ivanov et al. 2010). Rather, the efficiency of the first 48—hour extraction was estimated by calculating the proportion of individuals and species collected after the first extraction relative to the total number of individuals and species present after a 96—hour extraction.

Analyses of similarity (ANOSIM) was used to test for differences in the composition of ant assemblages between treatments and between the first and second Winkler extractions. The ANOSIM test is a non—parametric permutation procedure applied to similarity matrices. It produces a global R—statistic, which represents an absolute measure of distance between groups. When the R—value is close to 1, groups are highly distinct; whereas when the R—value is close to 0, groups are strongly similar (Clarke and Gorley 2006). Abundance data were fourth—root transformed prior to analyses to reduce the weight of common species. Similarity matrices were built using Bray—Curtis similarity measures. Tests were performed with the PRIMER v.6.1.6. software (PRIMER-E Ltd., www.primer-e.com). Other analyses were carried out using the SigmatStat v.2.03 software (Systat Software Inc., www.systat.com).

Our protocol was designed to answer to two independent, although complementary, questions. The first one is methodological and aims to evaluate the impact of leaf litter moisture on the efficiency of the Winkler method for extracting ants. The second is ecological and is about understanding the impact of an extended drought *per se* on ant assemblages. Here, we focused on the first question; differences between ant assemblages from rainfall—excluded and rainfall—allowed plots will be discussed in detail elsewhere (Delsinne et al. in prep.). Voucher specimens were deposited at the Royal Belgian Institute of Natural Sciences, Brussels, Belgium and at the “Universidad Técnica Particular de Loja”, Loja, Ecuador.

## Results and Discussion

The leaf litter samples from rainfall—excluded plots were on average 43% drier than samples from rainfall—allowed plots (Table 1). In total, 5649 ant specimens and 28 species were collected (Tables 1 and 2). Doubling the Winkler extraction time allowed the collection of 7.8 and 23.5% of supplementary individuals for rainfall—excluded and rainfallallowed samples, respectively (Table 1; Mann—Whitney Rank Sum Test, *p* < 0.01). For samples collected under rainfall—excluded plots (*n* = 60), the proportion of added individuals increased significantly with increasing moisture content of the leaf litter sample; both variables were arcsin square root transformed prior to linear regression analysis (*p* < 0.01; Figure 1). Adding either the volume or the weight (both log10 transformed) of the leaf litter into the model did not significantly improve the ability of the equation to predict the proportion of added individuals (arcsin square root transformed) (stepwise regression). Interestingly, when the three very wet samples (moisture content = 100 %; asinsqrt (100) = 1.57; Figure 1) were excluded from the analysis, the significance of the trend disappeared; the best—fitting equation of the regression analysis became: asinsqrt (Proportion of added individual) = 0.0316 + 0.248 × asinsqrt (Leaf litter moisture); adjusted r^2^ = 0.042, *p* = 0.068). These results indicated that the moisture content of the leaf litter sample significantly affected the efficiency of the Winkler method to extract ant individuals, at least when the moisture content was very high. The wetter the leaf litter, the longer the extraction should ideally last in order to collect all the specimens present within the sample. More data are needed to accurately estimate (1) the moisture content, above which it would be useful to extend the Winkler extraction, and (2) the duration of the extraction necessary to achieve similar extraction efficiencies.

Fortunately, the standard 48—hour extraction was sufficient to provide a reliable estimation of the composition and species richness of the ant assemblage, even when based on very wet samples. Indeed, there were no significant differences in the composition of the ant assemblage between Winkler extraction times (R = —0.333; *p* = 1 for both treatments; anosim tests). Moreover, the proportion of species added was not significantly different between samples from the two treatments (Table 1; Mann—Whitney Rank Sum Test, *p* = 0.395). Only three and eight samples, containing between one and six species after the first extraction, had one supplementary species documented after the second extraction for rainfall-excluded (*n* = 60) and rainfall-allowed plots (*n =* 60), respectively. At the treatment level, all the species collected after a 96—hour extraction were already documented after the first 48—hour extraction. Because ants are social insects, it is generally recommended to work with occurrence rather than abundance data (Longino 2000). Our results suggested this also limits biases caused by the leaf litter moisture.

The ant species rank—abundance curves based on 48—hour and 96—hour extracted samples were very similar for both treatments (Spearman Rank Order Correlations; for rainfall—excluded plots: n = 24 species; r = 0.990, *p* < 0.01; for rainfall—allowed plots: *n* = 20 species; r = 0.984; *p* < 0.01). Thus, doubling the extraction time did not substantively change the shape of the species relative abundance curve obtained after a standard 48—hour extraction.

There were no significant differences in the composition of the ant assemblage between treatments (R = 0.296; *p* = 0.2, anosim test based on the 48—hour Winkler extraction). At the species level, changes in relative abundance between rainfall—excluded and rainfall-allowed plots (Table 2) may be caused, for instance, by specific differences in drought tolerance. Nevertheless, it is possible that some individuals stuck to the wet litter of rainfall—allowed samples and were lost during the sifting process. This is suspected to be especially true for small ants, such as *Brachymyrmex* and *Solenopsis* species, since they are more prone to stick to wet litter. As a result, at least part of the differences in species relative abundance between treatments may be caused by the sampling procedure itself.

The few studies that have investigated the Winkler extraction efficiency for different periods of time demonstrated that a large proportion of both ant specimens and species were rapidly extracted from the samples (Ward 1987; Beshaw and Bolton 1994; Krell et al. 2005; Delsinne et al. 2008; Ivanov et al. 2010). For instance, a 48—hour extraction of samples from the Brazilian Atlantic rainforest allowed documentation of 85 and 95% of ant individuals and species, respectively (J.H.C. Delabie pers. comm.). Moreover, based on the analysis of 110 tropical and temperate assemblages collected with Winkler samples but with an extraction period varying from 10 to 72 hours (mean — SD: 32.3 ± 21.1 hours; median: 24 hours), Ward (2000) found that the extraction period had no significant effect on several measures of diversity such as species richness. Relatively short extraction times seem therefore justified when focusing on ants. Because the moisture content of the leaf litter only slightly decreased during the Winkler extraction (e.g., Sakchoowong et al. 2007; Delsinne pers. obs.), it is probable that the ant fauna migration out of the leaf litter relies mainly on the disturbance of the habitat rather than on its passive dessication.

In conclusion, a 48—hour Winkler extraction duration, as proposed for the A.L.L. protocol (Agosti and Alonso 2000), allows researchers to carry out reliable comparisons of leaf litter ant assemblages. Absolute abundance may be slightly underestimated when the moisture content of the leaf litter sample is high (e.g., ≥ 80%), but the assemblage structure (i.e., species richness, composition, and relative abundance) is correctly documented.

**Table 1.**
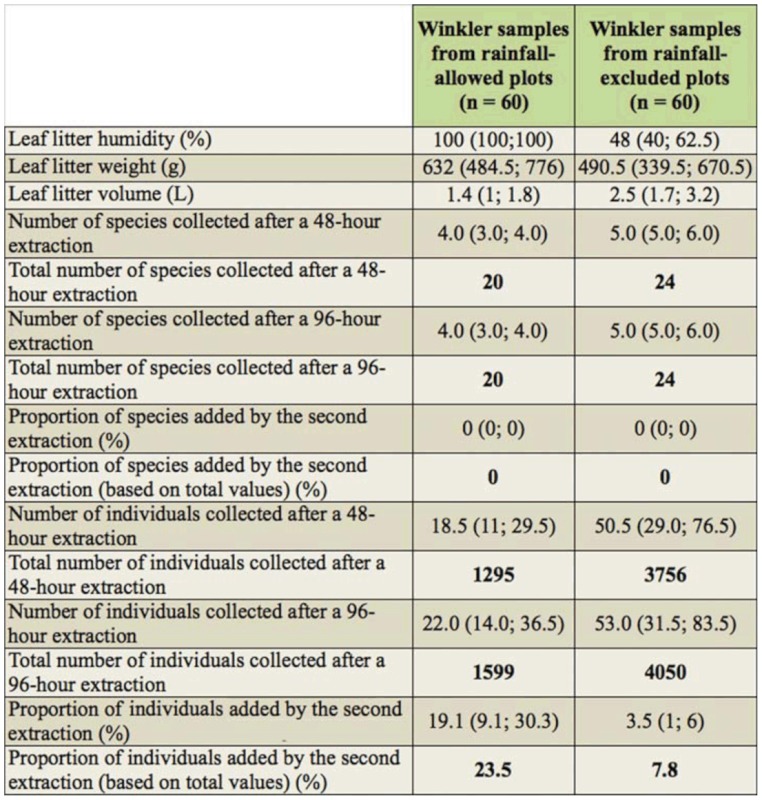
Leaf litter sample properties and efficiency of the ant fauna extraction for relatively dry and wet Winkler samples from rainfall—excluded and rainfall—allowed plots, respectively. Data are medians, interquartiles between parentheses, total values in bold.

**Table 2.**
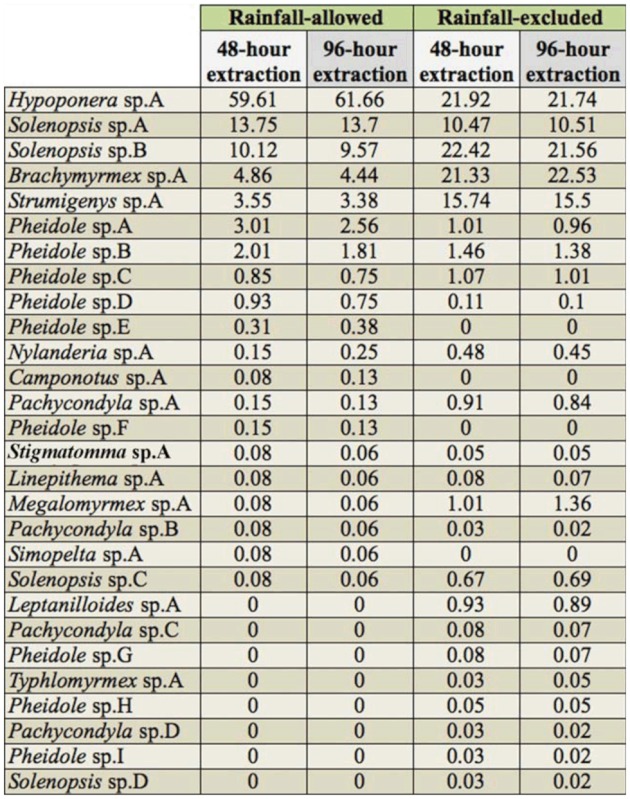
The 28 morphospecies collected and their relative abundance (%) for Winkler samples from rainfall—allowed and rainfall-excluded plots. Data from the 48—hour and the 96—hour extractions were computed separately.

## Figures and Tables

**Table:**
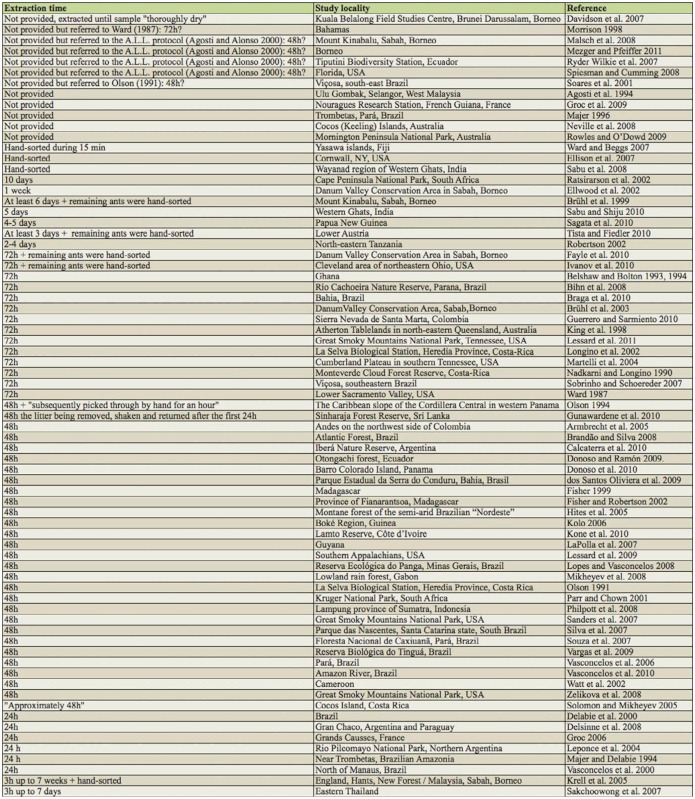
Winkler extraction duration used in published ant surveys. In December 2010, keywords such as “ants + Winkler”, “Formicidae + Winkler” and “A.L.L. protocol” were used to search studies dealing with ant diversity, ecology and biogeography on Web of Science and Google Scholar. Ant taxonomical studies were not included because their aim is not to compare ant assemblages in a standardized way. Where the same data set was used in several papers, only one was listed. The study locality is given in order to show that extraction time was rarely selected according to where the sampling was carried out.
